# Virtual facilitation best practices and research priorities: a scoping review

**DOI:** 10.1186/s43058-024-00551-6

**Published:** 2024-02-16

**Authors:** Asya Agulnik, Derrecka Boykin, Denalee M. O’Malley, Julia Price, Mia Yang, Mark McKone, Geoffrey Curran, Mona J. Ritchie

**Affiliations:** 1 Department of Global Pediatric Medicine, St. Jude Children’s Research Hospital, Memphis, TN USA; 2 Division of Critical Care, St. Jude Children’s Research Hospital, Memphis, TN USA; 3 Center for Innovations in Quality, Effectiveness and Safety, Michael E. DeBakey VA Medical Center, Houston, TX USA; 4 Menninger Department of Psychiatry and Behavioral Sciences, Baylor College of Medicine, Houston, TX USA; 5 Department of Family Medicine and Community Health, Rutgers Robert Wood Johnson Medical School, Research DivisionNew Brunswick, NJ USA; 6 Center for Healthcare Delivery Science, Nemours Children’s Health , Wilmington, DE USA; 7 Department of Pediatrics, Sidney Kimmel Medical College at Thomas Jefferson University, Philadelphia, USA; 8 Department of Internal Medicine, Section of Gerontology and Geriatric Medicine and the Sticht Center for Healthy Aging and Alzheimer’s Prevention, Wake Forest University School of Medicine, Atrium Health Wake Forest Baptist, Winston Salem, NC USA; 9 Coy C. Carpenter Library, Wake Forest University School of Medicine, Atrium Health Wake Forest Baptist, Winston-Salem, NC USA; 10 Department of Pharmacy Practice, University of Arkansas for Medical Sciences, Little Rock, AR USA; 11 VA Behavioral Health Quality Enhancement Research Initiative (QUERI), Central Arkansas Veterans Healthcare System, North Little Rock, AR USA; 12 Department of Psychiatry and Behavioral Sciences, College of Medicine, University of Arkansas for Medical Sciences, Little Rock, AR USA

**Keywords:** Implementation facilitation, Practice facilitation, Virtual facilitation, Implementation strategy, Scoping review

## Abstract

**Background:**

Facilitation is an implementation strategy that supports the uptake of evidence-based practices. Recently, use of virtual facilitation (VF), or the application of facilitation using primarily video-based conferencing technologies, has become more common, especially since the COVID-19 pandemic. Thorough assessment of the literature on VF, however, is lacking. This scoping review aimed to identify and describe conceptual definitions of VF, evaluate the consistency of terminology, and recommend “best” practices for its use as an implementation strategy.

**Methods:**

We conducted a scoping review to identify literature on VF following the PRISMA-ScR guidance. A search of PubMed, Embase, Web of Science, and CINAHL databases was conducted in June 2022 for English language articles published from January 2012 through May 2022 and repeated in May 2023 for articles published from January 2012 through April 2023. Identified articles, including studies and conference abstracts describing VF, were uploaded into Covidence and screened independently by two reviewers. Data extraction was done by two reviewers in Microsoft Excel; additionally, studies were evaluated based on the Proctor et al. (2013) reporting guidelines for specifying details of implementation strategies.

**Results:**

The search strategy identified 19 articles. After abstract and full-text screening, eight studies described by 10 articles/abstracts were included in analysis. Best practices summarized across studies included (1) stakeholder engagement, (2) understanding the recipient’s organization, (3) facilitator training, (4) piloting, (5) evaluating facilitation, (6) use of group facilitation to encourage learning, and (7) integrating novel tools for virtual interaction. Three papers reported all or nearly all components of the Proctor et al. reporting guidelines; justification for use of VF was the most frequently omitted.

**Conclusions:**

This scoping review evaluated available literature on use of VF as a primary implementation strategy and identified significant variability on how VF is reported, including inconsistent terminology, lack of details about how and why it was conducted, and limited adherence to published reporting guidelines. These inconsistencies impact generalizability of these methods by preventing replicability and full understanding of this emerging methodology. More work is needed to develop and evaluate best practices for effective VF to promote uptake of evidence-based interventions.

**Trial registration:**

N/A.

**Supplementary Information:**

The online version contains supplementary material available at 10.1186/s43058-024-00551-6.

Contributions to the literature

- Virtual facilitation (VF), or the application of facilitation using primarily video-based technology, is an emerging implementation strategy that has not yet been thoroughly described in the literature.
- In this scoping review of VF, we identify significant variability in how VF is reported and describe best practices for optimizing VF impact.
- Identified areas of needed future work include more consistent reporting of VF details to allow replicability and further study into strategies to optimize effectiveness of VF to promote uptake of evidence-based interventions.

## Background

Implementing evidence into clinical practice, a dynamic and multifaceted process that occurs in complex systems, is challenging. Facilitation, defined as “a process of interactive problem solving and support which occurs in the context of a recognized need for improvement and a supportive interpersonal relationship,” [1, 2] can address these challenges and support implementation of evidence-based practices and programs (EBPPs) [3–7]. Facilitation is most often applied within a multi-faceted strategy that bundles several strategies to support EBPP adoption, implementation, adaptation, and sustainment [8].

Facilitators, or the individual(s) responsible for supporting implementation, can be external to the organization implementing the innovation, internal to it, or both. Strategies they employ include stakeholder engagement, assessing current practices and potential barriers and facilitators to implementation, performing audits and providing feedback, and collaboratively developing implementation plans [9, 10]. Especially for external facilitators, building relationships with local site personnel and leaders is key to the facilitation process. Until recently, external facilitators conducted in-person site visits to begin these processes and establish relationships, with follow-up communications conducted remotely via telephone and email.

Since 2020, the coronavirus disease 2019 (COVID-19) pandemic challenged traditional implementation, including interrupting in-person site visits. Thus, facilitation pivoted to a fully remote, technology-enabled model [11]. Understanding how to build relationships with site personnel and engage them in the implementation processes using virtual facilitation (VF) is vital to the success of implementation efforts. Thorough assessment of literature on VF, however, is lacking. This scoping review aims to identify and describe conceptual definitions of VF, how it has been operationalized, and outline “best” practices for its use as an implementation strategy.

## Methods

We conducted a scoping review to identify literature on VF, defined as the application of facilitation for supporting implementation of EBPPs using video-based conferencing technologies, e.g., Zoom, Teams, and video-telehealth platforms. Scoping reviews are conducted to identify and clarify key concepts, available evidence, and gaps in knowledge to inform practice, policy, and future research [12]. Such reviews are particularly helpful in emerging areas that lack previous reviews. Our multi-phase process was informed by established review frameworks [3, 4].

### Search strategy

“Implementation facilitation” and “practice facilitation” are commonly used terms to describe application of facilitation to support implementation of EBPPs [3, 13–15]. The term “virtual” describes the use of video-based technologies for communication [16]. Our search strategy thus consisted of “implementation facilitation” or “practice facilitation” and the term “virtual.” With the assistance of a medical research librarian (MM), we conducted a search of PubMed, Embase, Web of Science, and Cumulative Index to Nursing & Allied Health Literature (CINAHL) databases in June of 2022 for English language articles published from January 2012 through May 2022. We repeated the search in May of 2023 for articles published from January 2012 through April 2023 (see Appendix pg. 2–3 for full search strategy). The results were imported into EndNote, a reference management software package, for deduplication, and then into Covidence, a web-based systematic review software platform, for study selection.

### Study selection

We conducted a two-stage screening process. First, titles and abstracts were independently screened by two reviewers (AA, MY), and disagreements were resolved by a third reviewer (MJR). Two independent reviewers (AA, MY) then conducted full text review with disagreements reviewed and discussed by the whole study team. We included peer-reviewed studies or conference abstracts which described the application of external facilitation for supporting implementation of EBPP using virtual modalities. Articles were excluded if (1) there was no application of facilitation for supporting implementation, i.e., they did not specify a designated person to support implementation; (2) most of the facilitation interactions with sites were conducted by telephone and email, and they did not include use of video-based conferencing technologies; or (3) there was insufficient information about the application of facilitation or the use of video-based conferencing technologies to determine if VF was used. During full text review, we identified and added two additional articles that expanded on studies described by abstracts identified by the search.

### Data synthesis

Data extraction and synthesis were conducted in accordance with PRISMA-ScR guidance [17]. Data were extracted by study, rather than by article, into Excel and reviewed by two of three authors (AA, DB, MY). Extracted data included the following: the study design, practice setting, and details of how and why VF was used. This included a description of technology used for VF, if it was used as the only implementation strategy or part of a “package” with other strategies, and if any in-person activities were included.

Using the Proctor et al. guidelines for reporting implementation strategies, [18] we additionally evaluated whether each study described all components of VF. To encourage consistent and thorough specification and reporting, these guidelines recommend that strategies be named/labeled consistent with existing literature and operationally defined. Furthermore, the guidelines recommend that strategy descriptions include who enacted the strategy, actions involved in applying the strategy, target of those actions, strategy timing and dose, anticipated outcomes, and justification for its use. One co-author (JP) coded whether each study met these reporting recommendations with a second co-author (GC) review; discrepancies were discussed and resolved during meetings.

Following extraction, data was synthesized across studies to identify reporting gaps and recommendations for best practices. Categories for “best” practices were identified based on the United States Department of Veterans Affairs (VA) Behavioral Health Quality Enhancement Research Initiative’s (QUERI) Implementation Facilitation guide [19], which included a chapter on VF, with an “other” section added to capture emergent VF practices not covered by these categories. Data synthesis and conclusions were reviewed and approved by all authors.

## Results

**Fig. 1:**
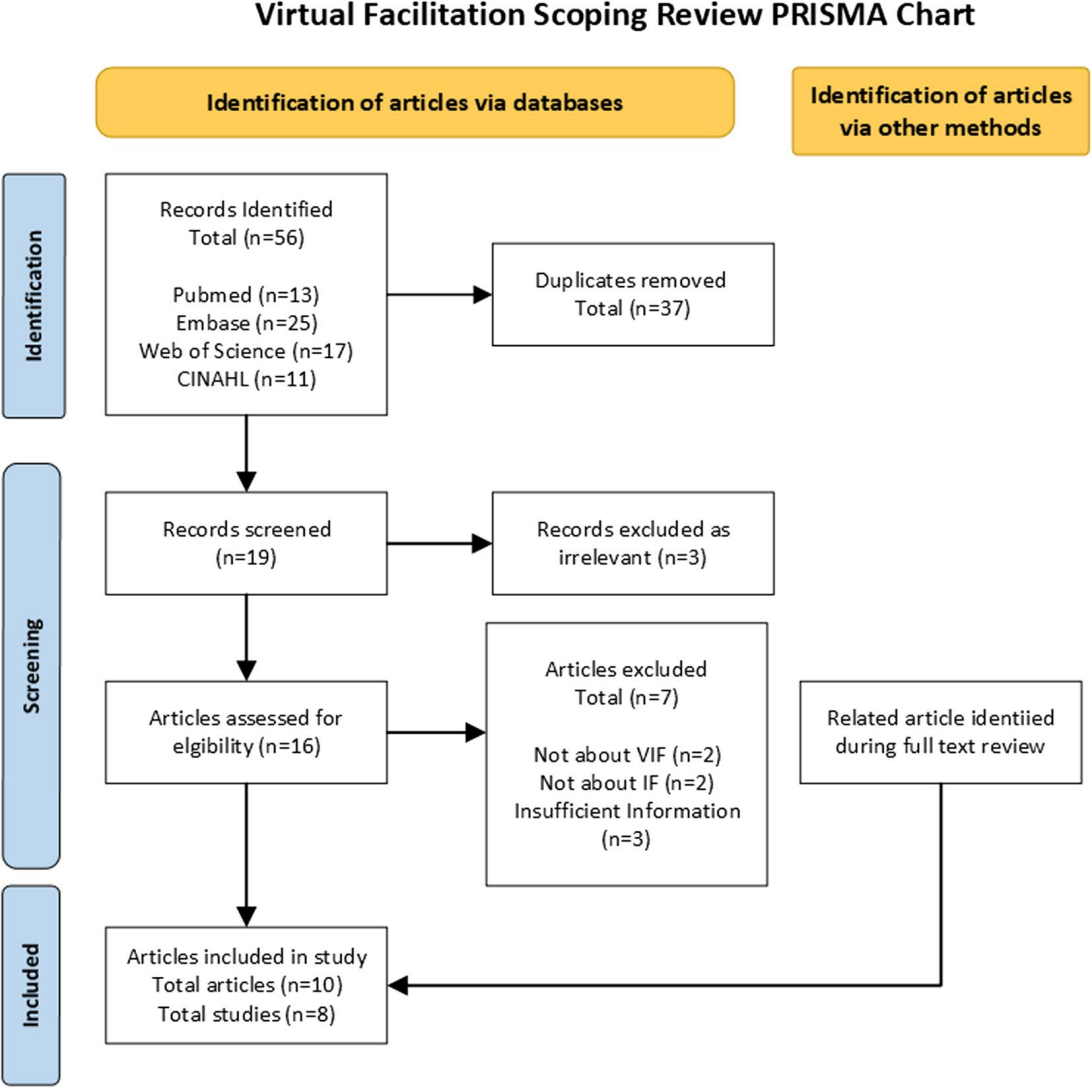
PRISMA. Adapted from: Page MJ, McKenzie JE, Bossuyt PM, Boutron I, Hoffmann TC, Mulrow CD, et al. The PRISMA 2020 statement: an updated guideline for reporting systematic reviews. BMJ 2021;372:n71

The search strategy identified 19 articles after removal of duplicates; 10 articles/abstracts representing 8 studies were included in analysis (Fig. 1). The use of VF across these 8 studies is summarized in Table 1. Details extracted included glossary of terms, why and how VF was used, and reporting of VF details, which are summarized below.

**Table 1:** Summary of included studies

Author	Title	Journal	Location	How VF was done	Glossary of synonyms	Theories, models or frameworks
Jortberg (2022) [20]	Making a Rapid Transition to Virtual Practice Facilitation: Tales of Facilitation During COVID-19	Annals of Family Medicine	Colorado, US	Virtual teleconferences	virtual practice facilitation	
Fox (2013) [21]	Improving evidence-based primary care for chronic kidney disease: study protocol for a cluster randomized control trial for translating evidence into practice (TRANSLATE CKD)	Implement Science	New York, US	Video-conferencing (GoToMeeting®), phone, and e-mail	Practice facilitation	TRANSLATE
Hartmann (2021) [16]	Virtual external implementation facilitation: successful methods for remotely engaging groups in quality improvement	Implement Science Communications	USA	n/a	Virtual external implementation facilitation	IHI breakthrough series
Lindsay (2017) [22]	Implementing Video to Home to Increase Access to Evidence-Based Psychotherapy for Rural Veterans	Journal of Technology in Behavioral Science	Mississippi, USA	Weekly teleconferences	Internal vs external implementation facilitation	PARIHS and RE-AIM
Smelson (2022) [23]	Testing implementation facilitation for uptake of an evidence-based psychosocial intervention in VA homeless programs: A hybrid type III trial	PLoS ONE	USA	Phone call and Skype	External implementation facilitation	Getting to Outcomes (GTO), i-PARIHS; ORCA
Ecker (2023) [24]	Feasibility of Group-Based Implementation Facilitation for Video Telemental Health	Journal of Technology in Behavioral Science	USA	Virtual group webinars	Implementation facilitation (IF), group facilitation, virtual facilitation	PIVOT approach
Malone (2021) [25]	A cluster randomized stepped-wedge trial to de-implement unnecessary postoperative antibiotics in children: the optimizing perioperative antibiotic in children (OPerAtiC) trial	Implement Science	USA	Webinars, virtual workshop, and phone calls	facilitation	IPARIHS and ERIC
Bednar (2023) [26]	Implementation and outcome evaluations of a multi-site improvement program in cancer genetics	Journal of Genetic Counseling	USA	Virtual teleconferences via WebEx, Doodle polls, Connect website, supplemented by 1 in-person meeting	Virtual implementation facilitation (VIF)	CDC Program Evaluation framework; IHI Model for Improvement

*Abbreviations*: *VF*, virtual facilitation

Questions:

- Prior in-person vs hybrid

- Urban vs rural vs multicenter geographically remote

### Glossary of terms

Among included studies, commonly used terms to describe VF included “virtual practice facilitation,” “virtual facilitation,” or “virtual external implementation facilitation.” Four of the eight included studies, however, did not label the facilitation strategy as virtual—these details had to be extracted from a detailed review of methods.

### Why VF was used

Specific justification for why VF was used included the COVID-19 pandemic [16, 20] or geographically distant location of centers in multisite studies [21, 26]. Notably, most studies did not explain why virtual, rather than in-person or hybrid, facilitation was selected as the implementation strategy.

### How VF was used

Included studies used a range of teleconferencing platforms to support VF, such as GoToMeeting, WebEx, and Skype. Additionally, one study [26] mentioned using Doodle polls to engage participants and a Connect website to allow networking across teams. Specific technology, however, was not reported in most (5) studies. Virtual meetings were frequently supplemented with telephone calls and emails to support ongoing communication between teams. One study (Bednar et al.) additionally described use of one in-person meeting as part of the primarily VF strategy.

### Reporting of VF methods

Results of application of the Proctor et al. guidelines are presented in Table 2. Reporting of specifications of VF use across studies was inconsistent and incomplete, with a range of zero to four missing specifications. Only three papers reported all or nearly all specifications. Justification for use of VF was the most frequently omitted specification.

**Table 2:** Were the Proctor et al. (2013) implementation strategy measurement reporting prerequisites met for virtual facilitation (VF)?

First author (year)	Study design	Practice setting	VF only vs. part of package?	Were Proctor reporting prerequisites met for virtual facilitation (VF)?								
				**Name VF?**	**Define VF?**	**Specify who provides VF?**	**Specify actions that comprise VF?**	**Specify target of VF?**	**Specify when VF used?**	**Specify dosage of VF?**	**Specify implementation outcome affected?**	**Specify justification for VF?**
Jortberg (2022) [20]	Qualitative	Primary care	–	Yes	Yes	–	–	–	–	–	–	Yes (COVID)
Fox (2013) [21]	Cluster RCT protocol	Primary care	Part of package	Yes	Yes	No	Yes	Yes	Yes	Yes	Yes	Yes (geographically remote sites)
Hartmann (2021) [16]	Description of best practices	N/A	VF only	Yes	Yes	No	Yes	Yes	No	No	No	Yes (COVID)
Lindsay (2017) [22]	Qualitative	VA; tele-health (rural)	Part of package	Yes	Yes	Yes	Yes	Yes	Yes	Yes	Yes	No (rural)
Smelson (2022) [23]	Hybrid III modified stepped-wedge trial	VA (suburban and rural) 2 VAMC and 7 homeless centers	VF only vs. VF + IF	Yes	Yes	Yes	Yes	Yes	Yes	Yes	Yes	No (suburban and rural)
Ecker (2023) [24]	Mixed methods	VA	VF only	No	No	No	Yes	Yes	No	No	Yes	No
Malone (2021) [25]	Protocol of cluster randomized stepped-wedge trial	Children’s hospitals (urban multiple centers)	VF only	No	No	Yes	Yes	Yes	Yes	Yes	Yes	No
Bednar (2023) [26]	Multi-site mixed methods program evaluation	Cancer centers (multiple urban centers)	Part of package	Yes	Yes	Yes	Yes	Yes	Yes	Yes	Yes	Yes (multiple centers)

*Abbreviations*: *IF*, in-person facilitation; *VF*, virtual facilitation; *–*, unknown due to only abstract available

### VF best practices

VF best practices were identified a priori by the study team based on published guidelines for implementation facilitation; synthesis across studies is described below.

### Stakeholder engagement

Development of a collegial, positive relationship between the facilitator and site stakeholders is central to success of VF as an implementation strategy. Multiple studies offered recommendations on strategies for engaging stakeholders during VF. These recommendations focused on practical methods for building relationships and structuring meaningful interactions between facilitators and stakeholders in virtual environments. For example, facilitators should work with stakeholders to identify effective mechanisms to promote timely communication and be vigilant of emails, telephone calls, and other messaging platforms (such as instant messages or text messages) to correspond with stakeholders in real-time. Additionally, videoconferencing calls should be planned in advance to account for potential technological failures, logistical challenges, and stakeholder disengagement.

In virtual environments, there is a greater need to foster a sense of “togetherness” among attendees. This can be accomplished by clarifying everyone’s role in meetings and promoting equitable participation [16]. Role playing can build team cohesion, communication skills, and conflict resolution [25]. Several studies highlighted the advantages of leveraging on-site internal facilitators and site champions to help manage local hierarchical structures and group dynamics [16, 21, 22, 24, 25]. Conducting group-based VF meetings with stakeholders from multiple sites (instead of one-on-one external facilitator-single site meetings) can maximize resources, especially whenever EBPPs needs to be rapidly deployed [24]. Other suggestions to optimize engagement included setting clear agendas distributed ahead of time, using visual aids, and creating structured handouts and easy-to-complete on-site activities that reinforce learning objectives, as well as breaking larger groups into smaller facilitated groups to promote meaningful discussions. Finally, several studies focused on the need to create opportunities for stakeholder feedback. Examples include audit and feedback procedures to discuss ongoing progress on implementation outcomes (e.g., adoption rates, patient encounters, intervention fidelity) with stakeholders [16, 21–23, 26]. Most studies emphasized that stakeholder interactions during VF should be tailored to the specific needs of each practice site [16, 20, 22].

### Understand recipient’s organization

To tailor the content and approach of VF to local context, facilitators must obtain relevant information about each site. Most studies addressed the need to tailor the intervention or approach to the recipient’s organization, such as the practice setting, context, and patient population [21–24, 26]. Strategies mentioned included using questions or surveys to better understand the facilitation recipient’s organizational culture and structure. Additionally, several studies included formal organizational readiness assessments [23, 25] or pre-implementation center-specific planning [26] and integrated these findings into their VF strategy.

### Facilitator training

Facilitators need to possess core skills and competencies (e.g., abilities to build relationships and create a supportive environment to transfer knowledge to support ongoing learning) [19] specific to VF. Examples of informal and formal training in VF were described in two studies. Hartman et al. added a weekly two-hour internal facilitator development training to existing team meetings and debriefing sessions led by expert consultants and focused on refining internal facilitators’ skills specific to VF. In Malone et al., external facilitators attended the VA Behavioral Health QUERI’s Implementation Facilitation Training and trained internal facilitators in VF. While other studies did not report whether facilitators received specialized training, increased reliance on technology to complete facilitation activities may require, at minimum, experience using video-based conferencing software and capacity to problem-solve emergent technological challenges.

### Facilitation piloting

Hartmann et al. emphasize the importance of piloting, evaluating, and refining VF processes to ensure successful implementation. They recommend using continuous quality improvement procedures (such as plan-do-study-act cycles) to identify best-fitting strategies and processes that match site-specific contexts. For example, external facilitators might experiment with different ways of giving and receiving feedback from stakeholders before deciding on a preferred mode of communication. Another example described having external facilitators receive feedback on visual aids from internal facilitators in advance of planned meetings to check for understanding.

### Facilitation evaluation

Several strategies were used to evaluate VF. One strategy involved having facilitators track the frequency, length, and types of interactions they completed, including email correspondence, videoconference calls, and other meetings [21, 23]. Validated measures can be used to measure aspects of VF [25, 26], such as acceptability, appropriateness, and feasibility [27]. Additionally, qualitative methods (e.g., field notes, semi-structured interviews) allow for detailed documentation of processes, decisions, and changes that occur during VF over time [20, 22, 25]. Mixed methods evaluations leverage the strengths of quantitative and qualitative methods to gain a richer understanding of how VF influences EBPP adoption [22, 25, 26]. Comparative studies are beneficial for evaluating the impact of VF on implementation outcomes (such as acceptability, adoption rates, number of patient encounters) relative to usual implementation or standard care practices [22, 23, 25]. Another evaluation approach was to assign external facilitators on the study team as “outside observers” to provide real-time objective input about factors influencing successful use of VF during meetings [16, 22]. Regardless of strategy, regular review of VF evaluation findings was deemed important for supporting continuous quality improvement processes [16] and providing timely feedback to stakeholders [20–22].

### Others

Several studies offered additional VF strategies not described in the categories above. For example, Fox et al. provided facilitators guidelines for the minimum number of meetings per center, along with structured virtual group review of reports on center progress in EBPP implementation. [21] Hartmann et al. emphasized the opportunity to “prioritize metacognition”—incorporating adult learning theory to allow participants to better learn new information through activities such as talking in groups, writing about what they learned, and reflecting on the facilitation session. [16] Uniquely, Bednar et al. integrated other virtual tools, such as polling and a dedicated website, to promote collaboration and interaction between team members beyond group meetings. [26] This study additionally integrated quality improvement training for local implementation teams using the Model for Improvement into VF sessions.

## Discussion

This scoping review evaluated available literature on use of VF as a primary implementation strategy, identifying best practices and current literature gaps. Our study found several discrepancies in how VF is reported, including inconsistent terminology, lacking details about how and why it was conducted, and limited adherence to published reporting guidelines. These challenges impact generalizability by preventing replicability and full understanding of this new methodology.

This scoping review identified 10 articles/abstracts describing 8 studies using VF as a primary implementation strategy. It is likely that this strategy is used more frequently in practice, especially since the start of COVID-19 in 2020. Surprisingly, in 2023, there remains relatively little literature on this topic. Beyond the pandemic, VF may offer unique advantages to in-person facilitation as a strategy to support implementation in rural and/or geographically remote sites [28], and this must be evaluated in future work. This review highlights an urgent need to increase research in this field with an emphasis on using consistent terminology and following established reporting guidelines [18] to support replication and comparison of effective VF strategies. This work should integrate best practices highlighted in this review, other strategies used during in-person facilitation such as engaging patients as stakeholders [19], and potentially novel approaches specific to this strategy.

In addition to recommendations for reporting on VF, this study identified several areas for future work. Grey literature [29–32] from professional conferences, abstracts, and fields outside of implementation science [19] are not fully integrated in this review and may offer additional guidance on VF strategies. The field is also lacking formal systematic reviews on facilitation and virtual EBPP delivery. Additionally, all included studies were conducted in the United States (US); thus, identified best practices may not be generalizable to other settings with variable broadband access [33–35]. Even within the US, infrastructure and technology access among practices sites will differ based on organizational, financial, geographic, and sociopolitical factors [36, 37]. Future work must center on digital equity and global scale in VF research.

Our study has several limitations. This focused scoping review targeted studies using VF as a primary implementation strategy. As such, we limited our search to terms of “implementation facilitation” and “practice facilitation” and English-language studies. Although these terms have been increasingly used, “facilitation” alone may also be used to describe this implementation strategy. Because this can also describe activities unrelated to supporting implementation, it was beyond the scope of our study to screen all articles using this term. We acknowledge that there may be studies using VF not included in our scoping review. Our focus, however, aimed to highlight the specific VF practice, and successfully identified major literature gaps and areas of future work for the field.

## Conclusion

This scoping review of VF as a primary implementation strategy identified significant variability on how VF is reported, preventing replicability and full understanding of this emerging methodology. More work is needed to develop and evaluate best practices for effective VF to promote EBPP uptake.

### Supplementary Information

**Additional file 1: Appendix 1.** Scoping review search strategy.

## Data Availability

Data collected for this systematic review are available on reasonable request through contacting the corresponding author (Dr. Agulnik, asya.agulnik@stjude.org).

## Abbreviations

CINAHL: Cumulative Index to Nursing & Allied Health Literature
COVID-19: Coronavirus disease 2019
EBPP: Evidence-based practices and programs
QUERI: Quality Enhancement Research Initiative
US: United States
VA: Veterans Affairs
VF: Virtual facilitation
