# Supplementary material for: Virtual facilitation best practices and research priorities: a scoping review
Source: Implement Sci Commun. 2024 Feb 16;5:16. doi: 10.1186/s43058-024-00551-6 (PMC10873989; doi:10.1186/s43058-024-00551-6)
Supplement: Supplementary file 1 — Additional file 1: Appendix 1. Scoping review search strategy. [file 43058_2024_551_MOESM1_ESM.docx]

**Appendix:**

**Virtual Facilitation Best Practices and Research Priorities: A Scoping Review**

Asya Agulnik*^1,2^, Derrecka Boykin^3,4^, Denalee M. O’Malley^5^, Julia Price^6,7^, Mia Yang^8^, Mark McKone^9^, Geoffrey Curran^10^, Mona J Ritchie^11,12^

1Department of Global Pediatric Medicine, St. Jude Children’s Research Hospital, Memphis, TN

2Division of Critical Care, St. Jude Children’s Research Hospital, Memphis, TN

3Center for Innovations in Quality, Effectiveness and Safety, Michael E. DeBakey VA Medical Center, Houston, TX

4Menninger Department of Psychiatry and Behavioral Sciences, Baylor College of Medicine, Houston, TX

5Department of Family Medicine and Community Health, Research Division, Rutgers Robert Wood Johnson Medical School, New Brunswick, NJ

6Center for Healthcare Delivery Science, Nemours Children’s Health System, Washington DC

7Department of Pediatrics, Sidney Kimmel Medical College at Thomas Jefferson University

8Section on Geriatric Medicine & Gerontology and the Sticht Center for Healthy Aging and Alzheimer’s Prevention, Department of Internal Medicine, Wake Forest University School of Medicine, Atrium Health Wake Forest Baptist, Winston Salem, NC

9Coy C. Carpenter Library, Wake Forest University School of Medicine, Atrium Health Wake Forest Baptist, Winston-Salem, NC

10Department of Pharmacy Practice, University of Arkansas for Medical Sciences, Little Rock, AR

11VA Behavioral Health Quality Enhancement Research Initiative (QUERI), Central Arkansas Veterans Healthcare System, North Little Rock, AR

12Department of Psychiatry and Behavioral Sciences, College of Medicine, University of Arkansas for Medical Sciences, Little Rock, AR

*Corresponding author:

Asya Agulnik, MD, MPH

Department of Global Pediatric Medicine, Division of Critical Care

St. Jude Children’s Research Hospital, Memphis, TN, USA

email: [asya.agulnik@stjude.org](mailto:asya.agulnik@stjude.org)

**Appendix 1. Scoping Review Search Strategy**

**PubMed**

| 1 | "Implementation facilitation" OR "practice facilitation" | 274 |
| --- | --- | --- |
|  |  |  |
| 2 | "virtual" | 99,796 |
|  |  |  |
| 3 | 2012:2023 [dp] | 13,945,960 |
|  |  |  |
| 4 | English [la] | 30,817,130 |
|  |  |  |
| 5 | #1 AND #2 AND #3 AND #4 | 8 |

**Embase**

| 1 | 'implementation facilitation' OR 'practice facilitation' | 285 |
| --- | --- | --- |
|  |  |  |
| 2 | 'virtual' | 467,448 |
|  |  |  |
| 3 | [2012-2023]/py | 18,275,045 |
|  |  |  |
| 4 | english:la | 36,671,324 |
|  |  |  |
| 5 | #1 AND #2 AND #3 AND #4 | 14 |

**Web of Science**

| 1 | TS=(“Implementation facilitation” OR “practice facilitation”) | 274 |
| --- | --- | --- |
|  |  |  |
| 2 | TS=(virtual) | 260,417 |
|  |  |  |
| 3 | PY=(2012-2023) | 30,624,120 |
|  |  |  |
| 4 | LA=English | 71,592,431 |
|  |  |  |
| 5 | #1 AND #2 AND #3 AND #4 | 4 |
|  |  |  |

**CINAHL**

| 1 | TI (“Implementation facilitation” OR “practice facilitation”) AND “virtual” OR AB (“Implementation facilitation” OR “practice facilitation”) AND “virtual” | **6** |
| --- | --- | --- |
|  |  |  |
| 2 | PY 2012-2023 | **5,015,640** |
|  |  |  |
| 3 | LA English | **8,601,840** |
|  |  |  |
| 4 | S1 AND S2 AND S3 | **6** |
